# Promoter Methylation and mRNA Expression of Response Gene to Complement 32 in Breast Carcinoma

**DOI:** 10.1155/2016/7680523

**Published:** 2016-03-29

**Authors:** Ebrahim Eskandari-Nasab, Mohammad Hashemi, Firoozeh Rafighdoost

**Affiliations:** ^1^Genetics of Non-Communicable Disease Research Center, Zahedan University of Medical Sciences, Zahedan 9816743463, Iran; ^2^Department of Clinical Biochemistry, School of Medicine, Zahedan University of Medical Sciences, Zahedan 9816743463, Iran; ^3^School of Dentistry, Zahedan University of Medical Sciences, Zahedan 9816743463, Iran

## Abstract

*Background*. Response gene to complement 32 (RGC32), induced by activation of complements, has been characterized as a cell cycle regulator; however, its role in carcinogenesis is still controversial. In the present study we compared* RGC32 *promoter methylation patterns and mRNA expression in breast cancerous tissues and adjacent normal tissues.* Materials and Methods*. Sixty-three breast cancer tissues and 63 adjacent nonneoplastic tissues were included in our study.* Design*. Nested methylation-specific polymerase chain reaction (Nested-MSP) and quantitative PCR (qPCR) were used to determine* RGC32 *promoter methylation status and its mRNA expression levels, respectively.* Results*. RGC32 methylation pattern was not different between breast cancerous tissue and adjacent nonneoplastic tissue (OR = 2.30, 95% CI = 0.95–5.54). However, qPCR analysis displayed higher levels of* RGC32* mRNA in breast cancerous tissues than in noncancerous tissues (1.073 versus 0.959; *P* = 0.001), irrespective of the promoter methylation status. The expression levels and promoter methylation of* RGC32 *were not correlated with any of patients' clinical characteristics (*P* > 0.05).* Conclusion*. Our findings confirmed upregulation of RGC32 in breast cancerous tumors, but it was not associated with promoter methylation patterns.

## 1. Introduction

Breast cancer is the most predominant cancer among females worldwide. Breast cancer and other malignancies result from stepwise genetic alterations of normal host cells and, possibly, from epigenetic changes in the behavior of not only malignant cells but also host cells that interact with the tumor, such as immune, vascular, and stromal cells [1, 2]. The term epigenetic refers to information which is transmitted from the parental genome to the next generation of cells which is not encoded by the primary DNA sequence. Epigenetic mechanisms are essential for the regulation of gene expression and genome integrity in normal cells [3]. Nearly 50% of the genes that cause familial forms of cancer undergo methylation-associated silencing in various sporadic forms of cancer, once they are mutated in the germ line [4].

The response gene to complement 32 (RGC32) protein, recently named chromosome 13 open reading frame 15, is a cell cycle regulator induced by activation of complements [5, 6]. Human* RGC32 is *found on the long arm of chromosome 13 and is mapped in the interval of 13q12–13q14 [7].

The* RGC32* gene product plays a dual role in both cell proliferation and tumor suppressor in certain types of cancers [8]. Deregulation of RGC32 expression has been detected in a large variety of human cancers. It is downregulated in high-grade prostate intraepithelial neoplasia, invasive prostate cancer, multiple myeloma, and drug-resistant glioblastoma, but upregulated in others, including cutaneous T cell lymphoma and ovarian and breast cancer [8, 9]. Overexpression of RGC32 leads to reorganization of cytoskeleton and promotion of cell migration, which could be an important mechanism of RGC32 in progression of cancer metastasis [10].

Epigenetic alterations such as DNA methylation regulate gene expression in normal mammalian development. However, promoter hypermethylation plays a chief role in cancer through transcriptional silencing of crucial growth regulators such as tumor suppressor genes [11]. Recently, Kim et al. have reported that* RGC32* is subjected to epigenetic silencing in non-small-cell lung cancers (NSCLCs). They found that the* RGC32* DNA methylation is associated with low or undetectable levels of RGC32 mRNA expression in malignant and corresponding nonmalignant lung tissues [5]. Their findings suggest that transcriptional inactivation of RGC32 expression may be caused by promoter methylation of that gene.

Therefore, given the proof that RGC32 functions as a tumor suppressor gene in certain types of cancers, in this study, we analyzed the promoter methylation status of* RGC32* and evaluated its correlation with its gene expression in breast cancer.

## 2. Material and Methods

### 2.1. Patients

This study included 63 breast paraffin-embedded tumor samples and 63 adjacent nontumor tissues from the same patients. The clinicopathologic characteristics of patients with breast carcinoma are summarized in Table 1. All breast specimens were reviewed by skilled pathologists. The inclusion criteria were female patient with primary breast cancer and the availability of the paraffin-embedded tissue along with patients' clinicopathologic data. Patients previously treated with neoadjuvant or adjuvant therapy as well as those missing clinicopathologic data, for example, HER2, ER, PR, and nodal status, were excluded. An informed consent was obtained from all subjects, and ethical committee of Zahedan University of Medical Sciences approved our study. DNA was extracted from tissues using the standard protocol by proteinase K treatment and salting-out extraction protocol as described previously [12, 13]. The quality and integrity of the DNA were checked by electrophoresis on 0.8% agarose gel, quantitated spectrophotometrically, and stored at −20°C till further use.

### 2.2. Sodium Bisulfite Modification and PCR Amplification of the RGC32 Promoter

The DNA samples were treated with sodium bisulfite, which converts unmethylated C to U. However, when the methylation occurs at the C residues, they will withstand the treatment. The protocol applied for bisulfite modification of DNA was described previously [11, 14] with major modifications. Concisely, to 10 *μ*L of DNA (approx. one *μ*g), NaOH solution was added to a final concentration of 0.3 M. Denaturation of the DNA strands occurred effectively after incubation of the mix at 50°C for 15 min. This mixture was then blended with 50 *μ*L of 2% low melting point (LMP) agarose and incubated at 50°C for 15 min. A 15 *μ*L drop of this mixture was pipetted into 300 *μ*L cold mineral oil (Sigma). The agarose/DNA drop quickly hardened in the oil and agarose beads shaped once they were incubated at −4°C for 30 min. Aliquots of 700 *μ*L of a 5 M bisulfite reagent (5 M sodium bisulfite, Merck; 125 mM hydroquinone, Merck; pH = 5.0) were added to each reaction tube containing a single bead. The tube was gently inverted to move the bead into the aqueous phase and was incubated at 55°C in a water bath for 4–18 hr under exclusion of light. Treatments were stopped by equilibrations against 1 mL of 1x TE (2 × 15 min) followed by desulphonation in 500 mL of 0.2 M NaOH (2 × 10 min). Finally, beads were washed with 1 mL 1x TE (Tris-EDTA) buffer followed by equilibrations against 1 mL of ddH2O (1 × 15 min). The beads were used directly for the PCR or kept at −20°C for several weeks without any loss of quality.

**Figure 1:**
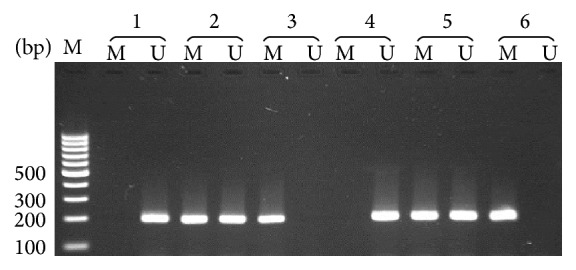
Methylation patterns of the* RGC32 *gene in breast carcinoma. “U” and “M” indicate unmethylated and methylated alleles, respectively. 1 and 4: UU; 2 and 5: MU; 3 and 6: MM.

Methylation status of the promoter region of* RGC32* gene was determined by a nested methylation-specific polymerase chain reaction (Nested-MSP), which boosts the sensitivity to detect the hypermethylated promoter by more than 50-fold. The primers used for the first stage of the MSP distinguish the bisulfite-modified template but do not discriminate methylated and unmethylated templates. Primers used for* RGC32* Nested-MSP were nested-forward (F): GGGTAAATATTTGGGGTTGTAAT, nested-reverse (R): TTCAACCCTACCAATCCCTTC; methylated-F: TCGCGGTTTTAGGGCGGGCGC, methylated-R: CCGCTCCCAACACGATCCGCG; unmethylated-F: TTGTGGTTTTAGGGTGGGTGT and unmethylated-R CCACTCCCAACACAATCCACA. The cycling conditions for the stage 1 of the Nested-MSP were 95°C for 10 min followed by 30 cycles of denaturation at 95°C for 15 s, annealing for 30 s at 60°C, extension at 72°C for 45 s, and final extension at 72°C for 10 min. The PCR product of the first stage (282 bp) was diluted 1 : 50 and subjected to the second stage of the Nested-MSP using two pairs of primers, one specific for the methylated alleles and another specific for the unmethylated as described previously by Kim et al. [5]. The PCR conditions for the second stage of the Nested-MSP were complete denaturation of DNA at 95°C for 10 min and 35 cycles involving denaturation at 95°C for 15 s, annealing for 30 s at 63°C, 45 s extension at 72°C, and final extension at 72°C for 10 min. The PCR products were verified on 2% agarose gels containing 0.5 *μ*g/mL ethidium bromide, and a photograph showing different methylation patterns was taken (Figure 1). The amplicons size for both methylated and unmethylated allele was 194 bp. To check the accuracy of our experiments, we repeated DNA methylation measurement randomly in 10% of samples. The methylation results were 100% concordant with the first results.

### 2.3. RNA Isolation, Preparation, and Real-Time PCR

Total RNA was isolated from formalin fixed paraffin-embedded tissue samples using a RNeasy® FFPE Kit according to the manufacturer's instructions. cDNA synthesis performed applying RevertAid*™* first strand cDNA synthesis kit (Fermentas) based on the manufacturer's procedure. Quantitative reverse transcriptase-PCR (qRT-PCR) for* RGC32 *was performed using the LightCycler ABI 7500 system and Maxima® SYBR Green/Rox (Fermentas). Specific primers for mRNA amplification of* RGC32* were used as previously described by Schlick et al. [15]. Reaction volumes of 20 *μ*L consisted of 10 *μ*M forward primer, 10 *μ*M reverse primer, 12.5 *μ*L Maxima SYBR Green/Rox, and 3 *μ*L of cDNA as PCR template. Gene expression was quantified by the comparative Ct method, normalizing Ct values to the housekeeping gene* GAPDH *and calculating relative expression values. The following program conditions were applied for qRT-PCR running: 95°C for 10 seconds followed by 40 cycles of 95°C for 10 seconds and 60°C for 1 min. Expression levels were normalized against* GAPDH*, which was amplified in the same run and following the same procedure described above. Gene expression was analyzed using 2^−ΔΔCT^ method. The primers sequences for Q-PCR analysis of* GAPDH (173 bp)* and* RGC32 (105 bp)* were GAPDH-F: TTGCCATCAATGACCCCTTCA and GAPDH-R: CGCCCCACTTGATTTTGGA; RGC32-F: AGCCTTCATTGCTGATCTTGA and RGC32-R: GCAGGTCCTCGGAACTTTCT.

### 2.4. Statistical Analysis

The statistical analyses of the data were done using the SPSS 18.0 software (SPSS Inc., Chicago, IL, USA). The association between methylation patterns was assessed by computing the odds ratio (OR) and 95% confidence intervals (95% CI) from logistic regression analyses.

Kruskal-Wallis one-way analysis of variance or one-way ANOVA test was used to assess possible association between methylation patterns and covariates in this study. *P* values below 0.05 were defined statistically significant.

## 3. Results

The promoter methylation status of the* RGC32* gene was examined on the DNA samples of 63 sporadic breast cancer tumors (average age: 46.2 ± 10.1 years) and 63 adjacent noncancerous tissues of the same patients. As presented in Table 2, the MM phenotype was more frequent in breast cancer tumors than noncancerous tumors (4.8% versus 0%), but no significant difference was found between two groups (OR = 2.30, 95% CI = 0.95–5.54). We also examined the* RGC32* methylation status in 63 blood samples of breast cancer patients and we found that all samples were unmethylated (data were not shown). Considering the impact of different covariates in the current study, we found no associations between* RGC32* promoter methylation and patients' age (*P* = 0.332), period age (*P* = 0.541), menopausal age (*P* = 0.197), tumor grade (*P* = 0.611), stage (*P* = 0.092), nodal metastasis (*P* = 0.245), estrogen receptor (*P* = 0.377), progesterone receptor (*P* = 0.489), and HER2 (*P* = 0.932).

**Figure 2:**
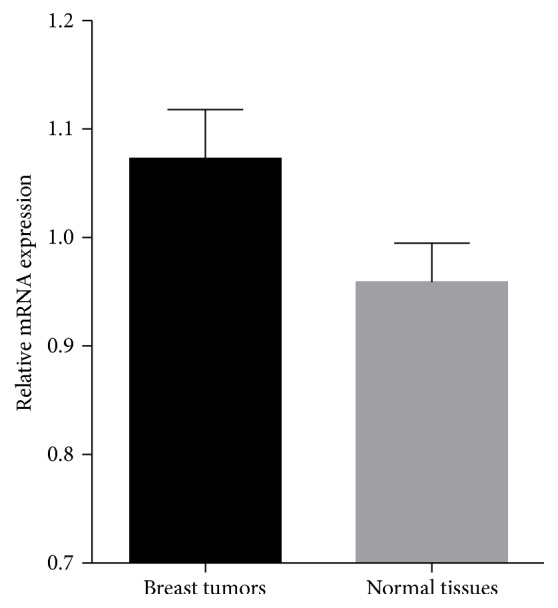
Relative mRNA expression of* RGC32* in breast cancerous and noncancerous adjacent tissues.

The qPCR analysis showed that the* RGC32* mRNA expression level was higher in breast cancerous tissues than in noncancerous breast tissues (*P* < 0.001). The ratios of GAPDH/RGC32 mRNA were 1.073 ± 0.045 and 0.959 ± 0.036 in breast cancerous and adjacent noncancerous tissues, respectively (Figure 2).* RGC32* expression level was neither associated with its DNA methylation pattern nor correlated to clinicopathologic characteristics of the patients (*P* > 0.05).

## 4. Discussion

In the current study, we found no significant difference between promoter methylation of* RGC32* gene in breast tumor tissues and adjacent nontumor tissues. However, the* RGC32* mRNA was overexpressed in breast cancer tissues compared to adjacent noncancerous tissues, but it was not associated with DNA methylation pattern of* RGC32* gene. Moreover, neither methylation pattern nor expression level of* RGC32* was associated with clinical characteristics of patients.

Concerning* RGC32* expression, our findings support the results of different studies reporting that RGC32 is upregulated in malignancies of ovary, lung, breast, and colon [8, 16]. Fosbrink et al. [16] localized RGC32 protein in various carcinomas including lung, colon, and breast and found that RGC32 is overexpressed in areas adjacent to the tumor carcinomas [8]. Similarly, our findings showed that RGC32 is overexpressed in breast tumors compared to adjacent nontumor tissues which highlights the plausible role of RGC32 in the tumorigenesis. In alignment with our finding, Vlaicu et al. have found that the RGC32 protein was absent from normal colon epithelial cells that were adjacent to the tumor that argues against its proposed tumor-suppressing role [8].

RGC32 plays a key role in modulating the activity of cell cycle-specific kinases, thus regulating cell cycle progression. Localized in the cytoplasm, RGC32 physically associates with cyclin-dependent kinase p34 CDC2 and plays a role in tumorigenesis and immunity [6–8]. Overexpression of RGC32 may promote cell replication by downregulating cell cycle inhibitors and contribute to the pathogenesis of malignancies, suggesting that RGC32 participates in tumor transformation and progression [6]. In addition, RGC32 plays an important role in regulating the acetylation of histones, a process that may possibly cause transcriptional activation and thereby support the possible function of RGC32 in tumor progression [17]. Vlaicu et al. [17] have suggested that RGC32 regulates the acetylation of histones H2B lysine 5 (H2BK5), H2BK15, H3K9, H3K18, and H4K8. Their findings proposed that RGC32 may be involved in the development of colon cancer by regulating chromatin assembly. Although growing proof points to a role for RGC32 in the promotion of cell proliferation, some studies have implicated RGC32 as a tumor suppressor. Kim et al. [5] have recently reported that* RGC32* is subjected to promoter hypermethylation and that its DNA methylation is associated with reduced expression of RGC32 in NSCLCs. In contrast, we did not find any difference in methylation pattern of* RGC32* promoter in cancerous and noncancerous breast tumors.

Regarding dual functions of RGC32 in a variety of carcinomas, it is speculated that these opposing reports could possibly arise from contradictory functions of RGC32 in different cell types. It is upregulated in cutaneous T cell lymphoma, colon, ovarian, and breast cancer and contributes to the cell proliferation and tumorigenesis, whereas it is downregulated in invasive prostate cancer, multiple myeloma, and drug-resistant glioblastoma functioning as a tumor suppressor [8].

Both global hypomethylation and regional hypermethylation have been studied in a wide spectrum of cancers [18], but CpG site-specific DNA methylation has recently been the center of attraction for cancer research. Recent analysis of DNA from different tissues demonstrated tissue-specific differentially methylated regions (DMRs) [19]. DMRs are genomic regions with diverse methylation statuses among multiple samples (tissues, cells, individuals, or others). They are regarded as probable functional regions involved in gene transcriptional regulation. The identification of DMRs among multiple tissues provides a comprehensive survey of epigenetic differences among human tissues [20, 21]. For example, these methylated regions that are distinctive to a particular tissue allow individuals to distinguish between tissue types, such as semen and vaginal fluid [22]. Additionally, DMRs between cancer and normal samples (C-DMRs) demonstrate the aberrant methylation in cancers and may affect the oncogenic process. Aberrant methylation of tDMRs has been reported in different types of cancer and may modify tumor suppressor genes and/or oncogenes [22]. Quantification of site-specific determination of CpG methylation is done by several approaches such as allele-specific bisulfite sequencing, bisulfite-pyrosequencing, and microarray-based genome-wide analysis. All these new techniques will improve our understanding of the pathophysiology of cancer [18, 23, 24].

Although all the previous studies have enabled a broader view of the genome-wide DNA methylation patterns, there still remain questions to be answered, for example, how the tDMRs are being established and what are the functions of gene-body tDMRs. Determining the human tDMR profile will not only provide important insights into the normal processes of tissue-specific differentiation; it may also identify markers of pathogenic processes, such as cancer.

To the best of our knowledge this is the first study that examined the association of* RGC32* promoter methylation and gene expression in breast carcinoma. The RGC32 mRNA expression level was much higher in breast carcinoma tissues than it was in nontumorous tissues, but it was not associated with methylation pattern of* RGC32* promoter. Additionally, we found that the* RGC32* promoter methylation was not different between breast tumorous tissues and nontumorous tissues, although the methylated phenotype was observed more frequently in tumorous tissues than in nontumorous breast tissues (4.8% versus 0). Meanwhile, we detected no promoter methylation in blood samples of the patients. All these findings suggest that* RGC32* DNA methylation is possibly abrupt in the cancerous tissues when compared to noncancerous tissues. There was one main limitation to this study. The small sample size of this study may have limited the statistical power of identifying the difference between groups. Therefore, larger sample size with in-depth analyses like bisulphite sequencing or CpG site-specific measurement can help in understanding the role of* RGC32* DNA methylation in breast cancer.

## Figures and Tables

**Table 1:** Clinical and pathological characteristics of breast carcinoma patients.

Characteristics	Cases *n* (%)
Tumor size (cm)	
≤2	18 (28.6)
2–5	44 (69.8)
>5	1 (1.6)
Number of positive nodes	
0	11 (25.0)
1–3	28 (65.9)
4–9	4 (9.1)
≥10	0 (0)
Histological grade	
I	6 (9.5)
II	16 (25.4)
III	11 (17.5)
IV	18 (28.6)
Unknown	12 (19.0)
Tumor stage	
I	10 (15.9)
II	31 (49.2)
III	14 (22.2)
IV	8 (12.7)
Histology	
Ductal carcinoma	56 (88.9)
Other	7 (11.1)
Estrogen receptor	
Positive	34 (54.0)
Negative	27 (42.9)
Unknown	2 (3.1)
Progesterone receptor	
Positive	21 (33.3)
Negative	42 (66.4)
HER2	
Positive	26 (41.3)
Negative	37 (58.7)

**Table 2:** Promoter methylation frequency of *RGC32* gene in breast cancerous and breast normal tissues.

*RGC32* methylation status	Normal tissues	Breast tumors	OR (95% CI)	*P* value
UU (%)	55 (87.3)	49 (77.8)	Ref.	—
UM (%)	8 (12.7)	11 (17.5)	1.54 (0.58–4.15)	0.390
MM (%)	0 (0)	3 (4.8)	(0.001)	0.999
Methylation				
Absent	118 (93.6)	109 (86.5)	Ref.	—
Present	8 (6.4)	17 (13.5)	2.30 (0.95–5.54)	0.090

UU: fully unmethylated promoter; UM: semimethylated promoter; MM: fully methylated promoter.
